# Improving Linkage to and Retention in Care in Newly Diagnosed HIV-Positive Patients Using Smartphones in South Africa: Randomized Controlled Trial

**DOI:** 10.2196/12652

**Published:** 2019-04-02

**Authors:** Willem Daniel Francois Venter, Alex Fischer, Samanta Tresha Lalla-Edward, Jesse Coleman, Vincent Lau Chan, Zara Shubber, Mothepane Phatsoane, Marelize Gorgens, Lynsey Stewart-Isherwood, Sergio Carmona, Nicole Fraser-Hurt

**Affiliations:** 1 Wits Reproductive Health and HIV Institute University of Witwatersrand Johannesburg South Africa; 2 World Bank World Bank Group Washington, DC United States; 3 National Health Laboratory Service Johannesburg South Africa

**Keywords:** cell phones, HIV, app, Africa, linkage to care, patient information

## Abstract

**Background:**

South Africa provides free antiretroviral therapy for almost 5 million people living with HIV, but only 71% of the eligible people are on treatment, representing a shortfall in the care cascade, especially among men and youth. Many developing countries have expanded access to smartphones; success in health apps raises the possibility of improving this cascade.

**Objective:**

SmartLink is a health app for Android smartphones providing HIV-related laboratory results, information, support, and appointment reminders to engage and link patients to care. This study aimed to evaluate the ability of SmartLink to improve linkage to care for HIV-positive smartphone owners.

**Methods:**

This study was a multisite randomized controlled trial in Johannesburg. The intervention arm received the app (along with referral to a treatment site) and the control arm received the standard of care (referral alone). Linkage to care was confirmed by an HIV-related blood test reported on the National Health Laboratory Service database between 2 weeks and 8 months after initiation.

**Results:**

A total of 345 participants were recruited into the study; 64.9% (224/345) of the participants were female and 44.1% (152/345) were aged less than 30 years. In addition, 46.7% (161/345) were employed full time, 95.9% (331/345) had at least secondary school education, and 35.9% (124/345) were from Zimbabwe. Linkage to care between 2 weeks and 8 months was 48.6% (88/181) in the intervention arm versus 45.1% (74/164) in the control (*P*=.52) and increased to 64.1% (116/181) and 61.0% (100/164) (*P*=.55), respectively, after the initial 8-month period. Moreover, youth aged 18 to 30-years showed a statistically significant 20% increase in linkage to care for the intervention group.

**Conclusions:**

Youth aged less than 30 years have been historically difficult to reach with traditional interventions, and the SmartLink app provides a proof of concept that this population reacts to mobile health interventions that engage patients in HIV care.

**Trial Registration:**

ClinicalTrials.gov NCT02756949; https://clinicaltrials.gov/ct2/show/NCT02756949 (Archived by WebCite at http://www.webcitation.org/6z1GTJCNW)

## Introduction

### Background

South Africa has the largest antiretroviral therapy (ART) program in the world, which provides free ART to approximately 4.4 million people living with HIV [1], and since its introduction in 2004, AIDS-related deaths and new HIV infections have been reduced by 58% and 46%, respectively [1]. The country strategy has been created in line with international guidelines and updated with the emergence of new bodies of evidence and global initiatives [2-4].

In 2015, the *90-90-90* initiative was introduced by the Joint United Nations Programme on HIV/AIDS and the World Health Organization as a way to further decrease new infections among the population, recognizing the large impact of ART on infectiousness, while also optimizing individual health. The initiative maximizes the effect of ART coverage by emphasizing that 90% of HIV-positive people should know their status, 90% of those eligible for ART should be initiated on ART, and 90% of those on ART should achieve and maintain viral suppression [5].

South Africa has accomplished moderate success with HIV testing and viral suppression, achieving 85% and 86% success rates, respectively; however, only 71% of the people eligible for ART are on treatment [6]. It is well documented that patients, especially young people aged less than 30 years and men, are being lost to follow-up along the entire HIV care cascade, but the most significant attrition is found during the stage from HIV diagnosis to the start of treatment [7-9]. Improving this deficit is needed to ensure that patients are initiated on ART early as patients lost during linkage to care often return as late presenters when they become seriously ill. Late presenters may also continue spreading the virus, further increasing the risk of infection and threatening the 90-90-90 targets [10].

In September 2016, South Africa adopted the *treat* *all* approach for ART treatment by dropping the CD4 thresholds for ART initiation completely [11], yet patients could still expect several clinic visits before initiating ART [12]. These visits consist of initial HIV testing, followed by determination of treatment eligibility, adherence counseling, and education, as well as baseline blood tests and a physical examination before receiving the antiretrovirals [12]. Each of these visits represents a risk to the continuum of care of the newly diagnosed HIV cases and simplifying this process has been hypothesized as a way to decrease patient drop-off. Various interventions such as home-based testing and treatment and same-day initiation of ART have been tested to address this attrition, but there remains a gap [10,12-14].

The emergence of mobile health (mHealth) in developing countries has enabled some successful interventions across the continuum of HIV care, especially on the promotion of treatment adherence. With 90% of the world’s population living in areas with mobile phone coverage and two-thirds of these people able to access data on their devices, mHealth provides an efficient method to engage the population [15]. Short message service (SMS) text messages and mobile apps have been used with moderate success in developing countries to improve ART adherence and appointment attendance [16-19]. South Africa has also experienced success with mHealth interventions, including the MomConnect program, which provides antenatal support through SMS and a help desk to almost 2 million pregnant mothers across the country [20].

The majority of mHealth interventions still focus on SMS text messaging, but by 2020, smartphone penetration in South Africa is expected to exceed 50% of the population [21]. The smartphones allow for data-based messaging, which should be considered for population scaling, as these platforms are much cheaper than SMS text messaging. Research surrounding linkage to care and the piloting, feasibility, and effectiveness of mHealth apps is needed to ensure that these interventions remain current as the population transition from basic phones to smartphones [15,19,22].

SmartLink is an mHealth app designed to provide HIV-positive smartphone owners with their laboratory results securely and rapidly, coupled with supportive information as well as prompts to link to care. Methods and information on the app development, including the challenges and limitations of the study, have been previously published [23] and will not be discussed in detail here.

### Objectives

This study presents the evaluation of SmartLink to improve linkage to care for newly diagnosed HIV-positive smartphone owners through a randomized controlled trial. Of particular interest is the linkage to care of men and youth aged less than 30 years, as these populations have been historically hard to reach with traditional interventions [7-9]. Virological suppression was also evaluated as a secondary outcome.

## Methods

### Trial Design

The study was designed as a multisite randomized controlled trial where newly diagnosed HIV-positive participants were approached upon having a positive HIV test and were then screened for trial eligibility. Eligible and consenting trial candidates were randomized 1:1 into either the intervention or the control arm of the study. Participants in the intervention arm were then aided with the installation and setup of SmartLink.

### Setting

The inner city of Johannesburg is one of South Africa’s most densely populated areas, with an estimated population of 1 million people; numerous socioeconomic challenges such as overcrowding, unemployment, crime, poverty, substance abuse, and sex work; and a high HIV prevalence [24]. The area has a well-established HIV testing and ART program, with some health care facilities providing ART to over 20,000 patients. However, the transient nature of the community makes it difficult to measure actual testing, linkage, and retention rates at the population level [25]. Participants were recruited at 5 public HIV testing sites (1 community health center, 3 clinics, and 1 tertiary hospital) from October 2015 to June 2016 and then followed up until February 2017.

### Participants

Trained field workers at the 5 testing sites approached newly diagnosed HIV-positive people for trial participation after they had blood drawn for CD4 count measuring. Trial candidates were prescreened. Participants were considered for the trial if they were a resident in the area, aged 18 years and above, not pregnant, and could read English or Zulu (2 commonly understood languages in the area) [23]. Individuals were then screened for app compatibility; ineligible participants were excluded from the study if they had no active subscriber identity module card in their phone, no Android smartphone, or no data on their phone. It was discovered that the app could not be installed if the participant had insufficient RAM on their phone or if their Android version was too old (pre-version 4.2), so these parameters were also added to the exclusion criteria. Eligible participants who passed screening were then recruited into the study and randomized 1:1 into the intervention arm or the control arm using a pregenerated randomization table.

### Intervention

Study staff assisted participants from the intervention arm with the installation of the SmartLink app, which was done with an Android install file and Wi-Fi dongle to allow installation at no data cost to the participants.

The app, available in English or Zulu, was designed to engage participants in their own care by directly providing them with 2 laboratory results; appointment reminders; and information about the laboratory tests, ART adherence, and HIV in general (Multimedia Appendix 1). The 2 laboratory results were CD4 count and viral load, and they were communicated in simple language. These values were also expressed visually on a color-coded scale that showed *normal* values and were accompanied by a short explanation of the results and guidance as to what action, if any, should be taken.

Participants randomized into the control arm received the standard of care, where participants received counseling and were referred to their local ART initiation site to collect their laboratory results and initiate appropriate treatment as needed. All participants, regardless of the study arm, were instructed to attend their local clinic for a follow-up within a few weeks of trial commencement and not to wait for the results on their phone.

### App Security

The SmartLink logo, app icon, and landing page made no reference to HIV, AIDS, or health care to ensure that a participant’s HIV or other health status would not be accidentally disclosed when viewing the app name or icon on a participant’s phone. Furthermore, to protect confidential medical information from being available to other people, app security was modeled after local banking apps. This ensured security and privacy by employing a username, password, and a personal identification number to gain access to personal health data.

### Outcomes

To capture HIV-related laboratory monitoring (our proxy for linkage to HIV care), evidence of an HIV-related laboratory test result between 2 weeks and 8 months of participant recruitment was sought. Test results were available on the National Health Laboratory Service (NHLS) database, which covers all local public facilities (but not initiation by private general practitioners or workplaces, although these provide very limited access in terms of absolute numbers), and included CD4, viral load, or creatinine clearance. Clinic visits were tracked after the initial 8 months until the completion of the follow-up in February 2017 to see if any lag to linkage to care was present in either trial arm. The viral load results were also analyzed to determine if virological suppression was achieved as a secondary outcome.

Due to these abovementioned independent databases as well as analytical data from the app developers, consolidation of these data was required. The investigators implemented a method to keep track of trial participants and their laboratory results by creating a centralized universal study dataset. To ensure intervention fidelity, this dataset was continuously monitored and evaluated by researchers to identify any potential variances [23].

### Data Analysis

On the basis of the market research conducted in early 2015 at the study sites and a primary outcome measured as a second HIV-related laboratory test between 2 weeks and 8 months, a sample size of at least 1000 participants for each study arm was anticipated to measure a 20% difference in linkage to care between the intervention and control arms of each study subgroup such as young men. This was calculated based on a significance of .05, a power of 80%, and an estimated loss to follow-up of 27% (Hillbrow Community health Centre data).

Descriptive statistics were used to summarize baseline characteristics, presented as categorical data with frequency (percentage). All outcomes were compared between the intervention and control arms by linkage to care with the Pearson Chi-square test for significance. All data analyses were performed with Stata version 12.1 (StataCorp LP, College Station, TX).

The SmartLink protocol was approved by the University of Witwatersrand’s Medical Human Research Ethics Committee (Certificate: M150606), the City of Johannesburg, and Gauteng’s Department of Health at the provincial level and was registered in ClinicalTrials.gov (NCT02756949).

## Results

### Participant Flow

**Figure 1:**
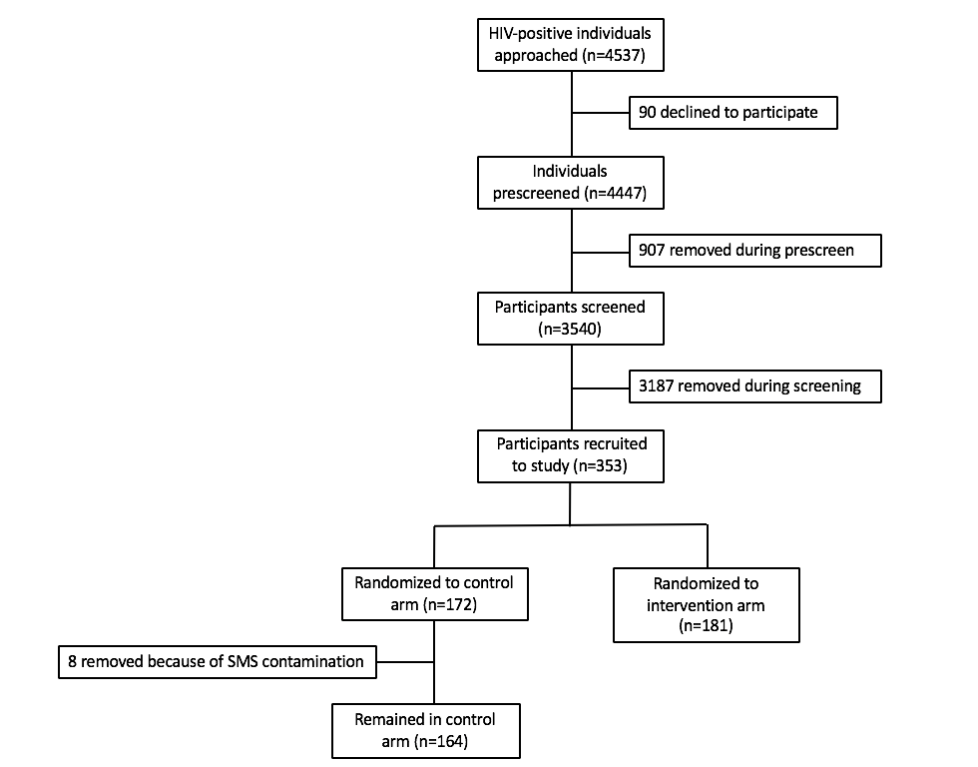
SmartLink participant flow diagram. SMS: short message service.

The participant flow diagram is shown in Figure 1. Of the 4537 individuals approached about the study, only 90 people (2.0%) declined to participate; however, a total of 4094 people (90.2%) were found to be ineligible during the prescreening and screening. The data from 8 participants in the control arm were also removed from analysis because of the erroneous sending of SMS reminders for their 6-month clinic appointment. Once removed, 164 participants (3.6%) remained in the control arm, and 181 participants (4.0%) remained in the intervention arm. A complete breakdown of enrollment based on inclusion and exclusion criteria has been reported [23].

**Table 1:** Baseline characteristics of SmartLink trial participants.

Characteristic		Control (n=164), n (%)^a^	Intervention (n=181), n (%)	Total (N=345),n (%)
**Sex**				
	Male	61 (37.2)	60 (33.1)	121 (35.1)
	Female	103 (62.8)	121 (66.9)	224 (64.9)
**Age (years)**				
	18-30	69 (42.1)	83 (45.9)	152 (44.1)
	31+	95 (57.9)	98 (54.1)	193 (55.9)
**Country** **of birth**				
	South Africa	95 (57.9)	103 (56.9)	198 (57.4)
	Zimbabwe	61 (37.2)	63 (34.1)	124 (35.9)
	Other	8 (5.9)	15 (8.3)	23 (6.67)
**Education**				
	Primary only	6 (3.7)	8 (4.4)	14 (4.1)
	Some secondary school	44 (26.9)	51 (28.2)	95 (27.5)
	Completed secondary school	85 (51.8)	98 (54.1)	183 (53.0)
	Attended/completed tertiary	29 (17.7)	24 (13.3)	53 (15.4)
**Employment status**				
	Employed full time	79 (48.2)	82 (45.3)	161 (46.7)
	Employed part time	22 (13.4)	37 (20.4)	59 (17.1)
	Unemployed	40 (24.4)	49 (27.1)	89 (25.8)
	Self-employed	16 (9.8)	10 (5.5)	26 (7.5)
	Student	7 (4.4)	3 (1.7)	10 (2.9)

^a^Total may not add to 100% because of decimal rounding.

### Baseline Characteristics

There were no significant differences in the baseline characteristics between the intervention and control arms (Table 1). Overall, only one-third of the participants were male (35.1%) and nearly half (44.1%) were youth aged less than 30 years. Almost half of the participants were employed full time (46.7%) and the majority had at least attended secondary school (95.9%). In addition, 57.4% of the participants were South African and just over one-third (35.9%) were from Zimbabwe. These baseline characteristics reflect the demographics of inner-city Johannesburg, where many migrants from Zimbabwe have settled and become part of the local population. These migrants are often well educated and possibly more likely to be employed than South Africans living in the inner city.

Although the 2 trial arms were well balanced in terms of participants’ characteristics, there was, however, an important bias as to which demographic groups entered the trial because of the smartphone eligibility criteria [23]. Those with less education, those earning less, and those unemployed or underemployed are less likely to have smartphones to be eligible for the study [23].

### Primary Outcome: Linkage to Care

This study called for a sample size of 2000 total participants; however, because of several challenges and limitations outlined, recruitment numbers were much lower than anticipated at 345 [23].

#### Total Cohort

Linkage to HIV care between 2 weeks and 8 months was just under 50% (45.1% control vs 48.6% intervention; *P*=.516) and increased to just over 60% (61.0% control vs 64.1% intervention; *P*=.551) after the initial 8-month period (Table 2).

#### Males

The male population, which was of specific interest, showed a slightly higher (but not statistically significant) linkage to care with the app between 2 weeks and 8 months (47.5% control vs 55.0% intervention; *P*=.412), but after 8 months, both these values were similar, approximately 66% (67.2% control vs 66.7% intervention; *P*=.949).

#### Youth Aged Between 18 and 30 Years

Despite the small sample size, a statistically significant difference was seen with youth aged between 18 and 30 years. Linkage to care between 2 weeks and 8 months was approximately 20% higher for youth with the app (31.9% control vs 53.0% intervention; *P*=.009), and this remained true after 8 months as well (50.7% control vs 69.9% intervention; *P*=.016; Table 2).

**Table 2:** Linkage to care.

Group		Linked to care for 2 weeks to 8 months						Ever linked to care					
		NLC^a^, n (%)		LC^b^, n (%)	Total, n	Pearson *χ*^2^	*P* value		NLC, n (%)	LC, n (%)	Total, n	Pearson *χ*^2^	*P* value
**Total cohort**													
	Control	90 (54.9)		74 (45.1)	164	0.4	.52		64 (39.0)	100 (61.0)	164	0.4	.55
	Intervention	93 (51.4)		88 (48.6)	181	0.4	.52		65 (35.9)	116 (64.1)	181	0.4	.55
	Total	183 (53.0)		162 (47.0)	345	0.4	.52		129 (37.4)	216 (62.6)	345	0.4	.55
**Males**													
	Control	32 (52.5)		29 (47.5)	61	0.7	.41		20 (32.8)	41 (67.2)	61	0.0	.95
	Intervention	27 (45.0)		33 (55.0)	60	0.7	.41		20 (33.3)	40 (66.7)	60	0.0	.95
	Total	59 (48.4)		63 (51.6)	121	0.7	.41		40 (33.1)	81 (66.9)	121	0.0	.95
**Females**													
	Control	58 (56.3)		45 (43.7)	103	0.1	.79		44 (42.7)	59 (57.3)	103	0.7	.40
	Intervention	66 (54.6)		55 (45.5)	121	0.1	.79		45 (37.2)	76 (62.8)	121	0.7	.40
	Total	124 (55.4)		100 (44.6)	224	0.1	.79		89 (39.7)	135 (60.3)	224	0.7	.40
**Youth aged between 18 and 30 years**													
	Control	47 (68.1)		22 (31.9)	69	6.8	.01		34 (49.3)	35 (50.7)	69	5.8	.02
	Intervention	39 (47.0)		44 (53.0)	83	6.8	.01		25 (30.1)	58 (69.9)	83	5.8	.02
	Total	86 (56.6)		66 (43.4)	152	6.8	.01		59 (38.8)	93 (61.2)	152	5.8	.02
**Aged over 30 years**													
	Control	43 (45.3)		52 (54.7)	95	1.9	.17		30 (31.6)	65 (68.4)	95	1.8	.18
	Intervention	54 (55.1)		44 (44.9)	98	1.9	.17		40 (40.8)	58 (59.2)	98	1.8	.18
	Total	97 (50.3)		96 (49.7)	193	1.9	.17		70 (36.3)	123 (63.7)	193	1.8	.18

^a^NLC: not linked to care.

^b^LC: linked to care.

**Table 3:** Viral load suppression.

Study group	Virally suppressed				
	Yes, n (%)	No, n (%)	Total, n (%)	Pearson *χ*^2^	*P* value
Intervention	28 (63.6)	16 (36.4)	44 (100.0)	0.2	.66
Control	23 (59.0)	16 (41.0)	39 (100.0)	0.2	.66
Total	51 (61.5)	32 (38.6)	83 (100.0)	0.2	.66

### Secondary Outcome: Viral Load Suppression

For participants who had viral load tests in the NHLS database, virological suppression was assessed as an outcome. As recruitment numbers were much lower than anticipated, participant results were also low, and no statistically significant results were reached; however, these values are presented for completeness (Table 3). As of February 2017, a total of 83 participants had viral load tests that could be used for analysis, 39 out of 164 (23.8%) from the control arm and 44 out of 181 (24.3%) from the intervention arm. With viral load suppression defined as less than 400 copies/mL, 59.0% of the control arm and 63.6% of the intervention arm experienced suppression; however, the *P* value of .663 negated any significance.

## Discussion

### Principal Findings

Although this was the first evaluation using a smartphone-enabled app to support linkage to HIV care in Africa, as far as we are aware, the study outcomes were limited due to being underpowered as a result of complications and limitations surrounding app compatibility. As a proof of concept, the SmartLink app worked as anticipated; however, the smartphone specifications required for installation excluded over 90% of candidates who volunteered to participate in the study. This is unfortunately a common trend in mHealth studies, where many interventions show generally positive results; however, they are often inconclusive or are not substantial enough when extrapolated out to a broader population or scaled up [16,26].

Although this study demonstrated that app-linked information and prompting can lead to increased linkage to care, the specific technology was not evaluated. The SmartLink app provided patients with laboratory results, information, and appointment reminders, but the relative efficacy of these specific components could not be explored. Despite the challenges in trial enrolment, one population of interest, youth aged 18 to 30 years, showed a statistically significant benefit of the app. This subpopulation experienced a 20% increase in linkage to care for the app group, and this is encouraging as HIV patients in this age group have historically been very difficult to engage with traditional interventions [8]. In South Africa, this population is 16% more likely to own a smartphone and 19% more likely to access the internet with their phone than their parents [27]. The high smartphone ownership coupled with our evidence of increased linkage in care strongly suggests that mHealth apps for engagement in care should be considered for this age group.

This demographic will become more and more familiar with technology, reinforcing the need to create a strong body of evidence surrounding these mHealth interventions. For children aged 9 to 17 years, 80% have access to internet on a smartphone and 84% own their own device. This generation is growing up with the internet, social media, and apps and already possesses the same mobile skills set as their parents, with children even surpassing them with knowledge about creating media and installing apps [28]. Future studies should focus on tailoring mHealth interventions toward youth, while also providing an opportunity to standardize counseling and support communications from health care providers [29].

### Conclusions

This proof-of-concept study has demonstrated that SmartLink can significantly increase linkage to care for youth aged 18 to 30 years; however, further evaluation with larger samples is required to recommend such an intervention for programmatic rollout. This research is of timely importance as demand for entry-level smartphones (sub-US $100) in developing countries had led to over 400 million smartphone units being sold in the first quarter of 2018 alone [30]. As smartphone penetration increases and prices decrease, new innovations such as using quick response code technology coupled with patient-held smartcards can allow for information to be transferred without internet access or data [31]. During this shift, mHealth apps should also be considered for incorporation into multifaceted interventions as bundling apps with SMS text messaging, phone calls, or in-person communications could be a way to optimally engage patients while app familiarity and technology continue to improve [32].

### Limitations

Secondary outcomes, such as ART initiation rates, feasibility, satisfaction, and participants’ knowledge, could not be evaluated because of the limitations, as outlined by Venter et al in 2018 [23]. Analytics on app use by participants also could not be evaluated because of complications in data collection between the devices and the back-end analytics software. Essentially, data exchange between the relevant systems could not be achieved during the trial, limiting the scope of log-in analytics to counts of app openings only. Finally, we acknowledge the limitations of the trial in terms of generalizability, as already mentioned. The eligibility criteria lead to a selected patient group. For instance, a relatively high proportion of Zimbabwean patients and more educated patients were better able to qualify for the trial.

## Appendix

Multimedia Appendix 1 Screenshots of the SmartLink app.

Multimedia Appendix 2 CONSORT-EHEALTH checklist (V 1.6.1).

## Abbreviations

ART: antiretroviral therapy
mHealth: mobile health
NHLS: National Health Laboratory Service
SMS: short message service
