# Supplementary figures and images for: Improving Linkage to and Retention in Care in Newly Diagnosed HIV-Positive Patients Using Smartphones in South Africa: Randomized Controlled Trial
Source: JMIR Mhealth Uhealth. 2019 Apr 2;7(4):e12652. doi: 10.2196/12652 (PMC6465976; doi:10.2196/12652)

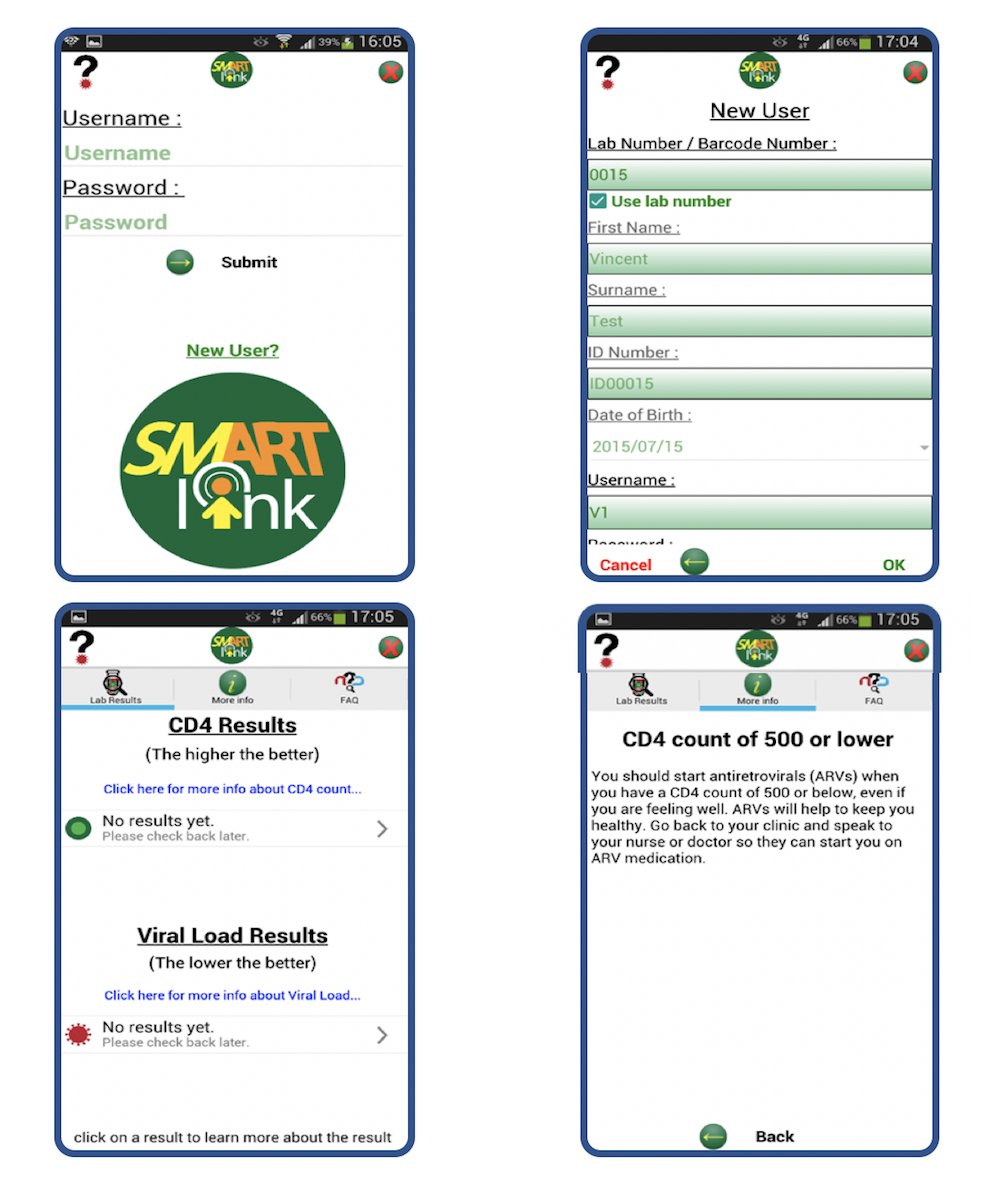

Supplement: Multimedia Appendix 1 [file mhealth_v7i4e12652_app1.png]
