# Supplementary material for: Improving Linkage to and Retention in Care in Newly Diagnosed HIV-Positive Patients Using Smartphones in South Africa: Randomized Controlled Trial
Source: JMIR Mhealth Uhealth. 2019 Apr 2;7(4):e12652. doi: 10.2196/12652 (PMC6465976; doi:10.2196/12652)
Supplement: Multimedia Appendix 2 [file mhealth_v7i4e12652_fig.pdf]

Publication Form for JMIR Publications Authors

If your manuscript has been accepted, please fill in this form, then print and sign. All authors need to sign section 2 and 3. Having them sign on separate forms is ok.
Please scan a copy of the signed form and upload to our management system (upload using the "Upload new Figure, Appendix or Supplementary file" functionality in the Editing tab and choose "License to publish" in the dropdown). If multiple authors across different institutions need to sign this, we suggest to sign the form electronically using resources like http://www.hellosign.com.
Questions? Check our Knowledge Base FAQ articles, e.g.
https://jmir.zendesk.com/hc/en-us/articles/115001384887
Your manuscript can only be published once we have the signed form on file.

Manuscripts for JMIR Publications are considered with the understanding that they have not been published previously in print or electronic format and are not under consideration by another publication or electronic medium.

The manuscript with the title

Do smartphones increase linkage to and retention in care in newly diagnosed HIV-positive patients in Johannesburg, South Africa: A multisite randomised controlled trial

authored by WD Francois Venter, Alex Emilio Fischer, Samanta Lalla-Edward, Jesse Coleman, Vincent Lau Chan, Zara Shubber, Mothepane Phatsoane, Marelize Gorgens, Lynsey Stewart-Isherwood, Sergio Carmona, Nicole Fraser-Hurt ("authors")

has been accepted for publication by JMIR Publications ("publisher"). A signed copy of this form must be on file with JMIR Publications before the manuscript can be published.

Manuscript #: 12652

Corresponding author: Alex Emilio Fischer

1. Publication Agreement

At least the corresponding author (preferably all authors) must sign the agreement. One author must be designated as the correspondent and his/her name, title of the manuscript, manuscript number, correspondent's address, and telephone and fax numbers must be included within this form. This part of the agreement is made between the corresponding author and JMIR Publications (50 Winners Circle, Toronto, ON, M4L 3Y7, Canada) or its legal successors.

1. I am authorized by my co-authors to enter into these arrangements.
2. I warrant, on behalf of myself and my co-authors, that:
   2.1 The article is original and has not been formally published in any other peer-reviewed journal and does not infringe any existing copyright or any other third party rights;
   2.2 I am/we are the sole author(s) of the article and have full authority to enter into this agreement and in granting rights to JMIR Publications are not in breach of any other obligation. If the law requires that the article be published in the public domain, I/we will notify the publisher at the time of submission upon which clauses 3 through 6 inclusive do not apply;
   2.3 The article contains nothing that is unlawful, libelous, or which would, if published, constitute a breach of contract or of confidence or of commitment given to secrecy;
   2.4 I/we have taken due care to ensure the integrity of the article. To my/our – and currently accepted scientific – knowledge, all statements contained in it purporting to be facts are true and any formula or instruction contained in the article will not, if followed accurately, cause any injury, illness, or damage to the user.

3. I/we retain copyright.
4. I/we grant to any third party, in advance and in perpetuity, the right to use, reproduce or disseminate the article in its entirety or in part, in any format or medium under a Creative Commons Attribution License (cc-by 4.0 http://creativecommons.org/licenses/by/4.0/) provided that no errors are introduced in the process, that JMIR Publications is duly identified as the original publisher, that proper attribution of authorship and correct citation details are given (including the URL to the original article on the JMIR or sister journals' websites), that a clear notice clarifying the license terms is provided (e.g. a notice saying that the article can be freely shared under a Creative Commons Attribution License), that the bibliographic details are not changed, and, if the work is reproduced or disseminated only in part, this fact is clearly and unequivocally indicated.

5. I/we grant to JMIR Publications (its successor and assigns) an irrevocable world-wide license for the full term of copyright in the article to publish it in any format or medium and identify itself as the original publisher and JMIR Publications as the original publisher in which the article first appeared.

6. In the event that the article is not published, these terms and conditions shall cease to apply and neither I/we nor the publisher shall have any further obligations towards the other in respect of the article or these terms and conditions, except for potentially outstanding article processing fees.

Signature(s):

Alex Fish

Signature of Corresponding Author

2. Letter of Responsibility

All authors should sign this statement:

I (we) have participated in the conception and design of this work and in the writing of the manuscript and take public responsibility for it. I (we) have reviewed the final version of the manuscript and approve it for publication. I (we) attest to the validity and legitimacy of the data in the manuscript and agree to be named as author of the manuscript.

Coauthors Name (in print)	Signature:
Nicole Fraser-Hurt	
ZARA SHUBBER	
MARELIZE GORGENS	
Samanta Lalla-Edward	
Alex Emilio Fischer	
WD Francois Venter	
Jesse Coleman	
Vincent Lau Chan	**Deceased**
Mothepane Phatsoane	
Lynsey Stewart-Isherwood	
Sergio Carmona	

3. Disclosure of Funding and Competing Interests

A description of sources of funding, financial disclosure and the role of sponsors must be included in the **Acknowledgement** section of the manuscript. This description should include:

- The involvement, if any, in review and approval of the manuscript of publication
- Role of sponsors

In addition, authors must disclose in a **Conflict of Interest** section if they have personal financial interests related to the subject matters discussed in the manuscript (if there are no conflicts, JMIR will print "None declared."). It is not unusual for JMIR Publications that authors are, for example, owners or employees of Internet/mhealth/EMR companies that market the services described in their manuscript. There is nothing wrong with this; but editors and readers must know about this, thus these facts must be disclosed.

I (we) certify that financial and material support for this research and work are completely disclosed in the **Acknowledgement** section.

I (we) warrant that I (we) have no further financial interests in the drugs, devices, software, computer programs, Internet companies, Internet service providers, or procedures described in the enclosed manuscript, except as those disclosed in the **Conflict of Interest** section of the manuscript. The **Conflict of Interest** section also contains all my affiliations with or financial involvement (e.g. employment, consultancies, honoraria, stock ownership or options, expert testimony, grants or patents received or pending, royalties) with any organization or entity with a financial interest in or in financial competition with the subject matter or materials discussed in the manuscript.

Coauthors Name (in print)	COI y/n (circle)	Signature:
Nicole Fraser-Hurt	y/n	
ZARA SHUBBER	y/n	
MARELIZE GORGENS	y/n	
Samanta Lalla-Edward	y/n	
Alex Emilio Fischer	y/n	
WD Francois Venter	y/n	
Jesse Coleman	y/n	
Vincent Lau Chan	y/n	Deceased
Mothepane Phatsoane	y/n	

Lynsey Stewart-Isherwood	y/n (n circled)	[signature]
Sergio Carmona	y/n (n circled)	[signature]
	y/n	

<u>If any of the coauthors has circled y, it is the responsibility of the corresponding author to ensure that appropriate language to disclose the COI is added to the final manuscript version (e.g. during copyediting or in the proofs), and the responsibility of the co-author to check the final galleys to ensure that the COI is accurately and completely disclosed.</u>

Thanks for publishing with JMIR Publications – The leading eHealth Publisher.

JMIR Publications
